# The Province of the Dentist

**Published:** 1860-11

**Authors:** J. Taft


### DENTAL REGISTER OF THE WEST.

VOL. XIV.] NOVEMBER, 1860. [No. 5.

### Original Essays and Communications.

### THE PROVINCE OF THE DENTIST.

BY J. TAFT.

ALMOST every one, at the presentation of this subject, will say; I understand this perfectly, I know what my duty is as a dentist. Notwithstanding this assertion, are there not many cases in which none know exactly what should be done? Perhaps all know that filling, extracting and inserting teeth, come within the legitimate province of the dentist; but is the best course always clear, in the details of the different divisions? In regard to the natural teeth, every one understands that it is the business of the dentist to wrest the teeth from the ravages of disease, and restore them to future usefulness. But how this is to be done in all cases, and under all circumstances, is a point that it may not be amiss to consider

We will give an example, illustrating the principles we wish to present:

A patient presents himself with a set of teeth well organized; but by neglect they have become much decayed, though not so much so, but that they may be efficiently filled, and thus saved for future usefulness, if the patient keeps them in a proper condition; but without this, they would certainly not be saved.

What is the duty of the dentist in such a case? Is it simply to fill the teeth, and let the patient go without anything farther, or without any instructions? or is it to do just what the patient directs, and nothing more, or should he explain all the circumstances and conditions as perfectly as possible to the patient, and then pursue the course the patient directs, and if the patient does not choose to pursue a proper course, would it be best to refuse to do anything at all?

Now, we consider it proper and legitimate, under all ordinary circumstances, to give information to the patient, upon all understandable points. Care and discrimination, however, must be exercised in accomplishing this; it should in all cases be done in such a manner as to exclude even the appearance of sinister motives. This will in some cases be a very delicate matter, and there are some cases in which it is worse than useless to attempt to give any information in regard to the teeth.

In the case referred to, there is a diversity of opinion, in regard to what should be done. If the patient puts himself entirely in the hands of the operator, and says, "Now, do everything that the welfare of my teeth requires, and that in the best manner, and give me such directions as you think proper, and I will obey them,"—in cases of this kind, there would certainly be no controversy in regard to the course to be pursued; but this class of patients is not very large. If a patient presents himself, and says he has two teeth decayed and wishes them filled, and directs the operator to them, and there are two others decayed, of which the patient is ignorant, that require attention as much as the first, is it the duty of the operator to fill the teeth to which his attention was first directed, and refrain from notifying the patient of the condition of the others? The operator would certainly fall far short of his duty, and he should be held responsible for the loss of those teeth. The dentist here occupies just the position, and is involved in the same kind of responsibility, though it may be not to the same extent, as the physician, who, called to treat a patient for a certain disease, finds another malady, of which neither the patient nor his friends were aware, equally as dangerous as the one of which they were cognizant, and as urgently requiring treatment, and refrains from acquainting them with the fact, and does not give them any treatment whatever. Every one can see that such a physician would be culpable, and no one would employ him a second time. No one would ever think of accusing him of sinister motives, for revealing the existence and danger of the hidden malady, no, not even were he to suggest immediate treatment.

We refer to this point in practice, because we have occasionally heard our professional brethren, and some of those occupying a high position too, make the declaration, that they never do anything more than they are requested by the patient to do in the outstart; that they never examine, nor refer to the other teeth, unless they are especially required to do so. Now, we consider it either a false delicacy or foolish dignity that prompts to this course. The presumption should always be where a patient presents himself with diseased teeth, that he comes to be restored to health, at least so far as the special malady is concerned, unless he expresses the desire that only so much as he describes shall be done. Then the question may arise with the operator whether he will do anything or not; for that which he may do, will be comparatively of little value, in consequence of the remaining affections at other points.

Now, we think the true course is very plain to any one desiring to perform his whole duty, and that is, not to urge the patient to have more done than he desires, but to make him fully aware of the true condition of his teeth, and then, if he does not know what should be done, suggest to him the proper course to be pursued, making him acquainted with all the circumstances and probabilities of the case, and from this point the patient makes the decision; and urging or pressing beyond this point, is wholly incompatible with true professional character.

A patient may present himself with teeth similar to the case given in the beginning, that have been, in regard to cleanliness, wholly neglected, and there is no probability that in the future they will be better cared for; he requests the teeth filled, but refuses to have them cleaned, and gives no promise for that particular in the future; in such a case, however good the teeth may have been originally, it is comparatively useless to fill them. Here we think the duty of the operator is clear; he should make the patient acquainted with the necessity for cleanliness, and that his own persevering effort will be required to accomplish it, that the welfare of the teeth demands it, his own well-being requires it, and that no filling, however skillfully and perfectly performed, will suffice to save the teeth, while they are the nuclei of an accumulation of filth. It is a question for each one to decide for himself, whether under such circumstances, he should do anything. It would probably be a legitimate presumption, that any one applying for treatment of carious teeth, would pursue that course, and employ those means, which would secure to him the greatest good from the operation performed upon them. Such care, however, is not always exercised. Some patients seem to think that when teeth have been operated upon by the dentist, that that care bestowed upon them ought to suffice for two or three years; "that when they have once been fixed, they are safe for a long time to come," and usually it will be found that the teeth of these persons have truly enough got in a fix. In such cases, where the teeth are of good structure, and the vitality strong, it is admissible to fill them, even though they be neglected afterwards, for the existence of the teeth would certainly be thereby prolonged; but in those teeth of frail structure and feeble vitality, unless they can be kept in good condition afterward, they should not be filled. This we think would be the proper course to pursue in general; there are probably exceptions, however.

Though it is, as a general rule, not advisable to fill teeth, and especially frail teeth, when they will not be cared for afterwards, there are other operations that may properly be performed under all circumstances, when requested by the patient. For example, if the teeth require cleansing, it is proper in all cases to do it, whether anything else, such as filling the decayed teeth, and extracting offensive ones, be performed or not, and even if there is little probability that they will be cared for in future. This is advisable because it is beginning at the right point, and starting in the right direction; it shows, at least for the time being, some due regard for the teeth, and the probabilities are that the patient will keep the teeth clean, and besides this, if the teeth are in an impure and filthy condition from deposits upon them, decay will go on far more rapidly, and disease of the gums and surrounding tissues will be liable to occur.

It is always proper to remove a diseased and offensive tooth, when desired by the patient, if it is beyond remedy, and though it may not have attained this point, if the patient will not have it restored to usefulness. It is right to remove one or two offensive teeth, though there be twenty others, whose removal is quite as important, that the patient will not permit to be touched; when these two teeth are removed, some good is accomplished, though twenty others remain, but in this instance, as in every other, it is the duty of the operator to endeavor to pursue that course which will secure to his patient the greatest good.

From this subject arises the question, should the dentist, under any circumstances, perform an operation that he knows will be of no real value, though he may be urged to it by the patient. No one with a true sense of duty will feel at ease, or that self-approval, which every honest man ought to feel, when he does that for a fee, which he knows will be of no value to any one, even though that one may not be aware of the fact; indeed, the true man would be self-reproved all the more, for having practiced deception upon his victim, as well as inflicted a worthless operation. There are still other reasons, that should forbid an operator performing worthless operations, even though urged by the patient. Operations always have an influence corresponding to their efficiency, and always, even under circumstances of that kind, the patient holds the operator responsible, and the probabilities are, that numerous other persons will become acquainted with the fact that the work is worthless. We think the position quite tenable, that no dentist is justifiable under any circumstances, in performing operations that will be of no real value , that by doing so, he does injustice to himself, to his patients, to the community, and to his profession, yea, to every member of it. This is not only true of those miserably worthless operations, that could be of no value under any circumstances, but it is also true of the most perfect and skillfully performed operations, when there is connected with them palpable circumstances, that render them necessarily inefficient.

